# MGF-Based Mutual Approximation of Hybrid Fading: Performance of Wireless/Power Line Relaying Communication for IoT

**DOI:** 10.3390/s19112460

**Published:** 2019-05-29

**Authors:** Zhixiong Chen, Cong Ye, Jinsha Yuan, Dongsheng Han

**Affiliations:** Department of Electronics and Communication Engineering, North China Electric Power University, Baoding 071003, China; YeCong@ncepu.edu.cn (C.Y.); 51550565@ncepu.edu.cn (J.Y.); handongsheng@ncepu.edu.cn (D.H.)

**Keywords:** hybrid fading, harmonic mean, Internet of Things (IoT), moment generating function (MGF), mutual approximation

## Abstract

Wireless and power line communications (PLC) are important components of distribution network communication, and have a broad application prospect in the fields of intelligent power consumption and home Internet of Things (IoT). This study mainly analyzes the performance of a dual-hop wireless/power line hybrid fading system employing an amplify-and-forward (AF) relay in terms of outage probability and average bit error rate (BER). The Nakagami-m distribution captures the wireless channel fading; whereas the PLC channel gain is characterized by the Log-normal (LogN) distribution. Moreover, the Bernoulli-Gaussian noise model is used on the noise attached to the PLC channel. Owing to the similarity between LogN and Gamma distributions, the key parameters of probability density function (PDF) with approximate distribution are determined by using moment generating function (MGF) equations, joint optimization of *s* vectors, and approximation of LogN variable sum. The MGF of the harmonic mean of the dual Gamma distribution variables is derived to evaluate the system performance suitable for any fading parameter *m* value. Finally, Monte Carlo simulation is used to verify the versatility and accuracy of the proposed method, and the influence of the hybrid fading channel and multidimensional impulse noise parameters on system performance is analyzed.

## 1. Introduction

With the rapid development of 5G mobile communication, the power system pays more and more attention to communication technology. The application of cutting-edge technologies such as big data, cloud computing, Internet of Things (IoT), mobile Internet and physical information fusion system in power systems has also attracted widespread attention. With the development of power supply networks, the coverage area of power lines has progressed considerably. The optimization of existing power supply network resources and the transmission of reliable information on power lines have gradually attracted the attention of researchers [1]. The power line serves not only as a medium for transmitting power but also for transmitting data for communication. Power line communications (PLC) can reach anywhere power lines exist, especially where the wireless signal is weak, such as underground, under water and metal-walled rooms. In communication architectures, PLC [2] uses the existing power line infrastructure to transmit information with power and provides cost-effective and extensive coverage of smart grid solutions [3].

However, the power line was originally designed for power transmission rather than specifically for communication. Therefore, problems such as insufficient reliability and inaccessibility of the mobile terminal exist in the PLC. First, the attenuation of long-distance and high-frequency signals significantly result in limited bandwidth. Second, the complicated noise, which is composed of background and impulse noises, in the power line is different from those of other communication channels. Third, the transmission power of the PLC is limited by electromagnetic compatibility rules. Finally, the PLC channel varies with location, network topology, and connection load. Therefore, this study considers the collaboration between PLC and other communication networks (such as wireless networks). When the mobile terminal encounters obstacles that lead to poor link quality, it can transmit via the power line channel to ensure the reliability of communication. In the absence of the power line or with a poor quality of PLC, wireless transmission can be performed to ensure the normal operation of communication [4]. The joint PLC/wireless dual-media cooperative communication technology has the practical advantages of integrating resources, complementary advantages, saving construction cost and improving overall system performance. In fact, the concept of hybrid systems has been discussed since 2005. The latest research results include multipath transmission [3,5], relay forwarding [6,7,8,9,10,11,12,13], multimedia collaboration [14,15,16,17,18], parallel communication [15,17] and other PLC collaboration technologies.

For the cooperation technology of wireless and PLC, extensive research, such as [6,7], uses the relay cooperation scheme of PLC and wireless communication because the direct link cannot meet the requirements of communication in many cases, and the relay collaborative technology can resist fading and improve the communication links. Research on relay is mainly divided into dual-hop [8,9] and multihop [10,11] relays, with both focusing on decode-and-forward (DF) and amplify-and-forward (AF) relay protocols. In terms of power communication, literature [11] analyzes the performance of multihop PLC systems by using the DF relay under Log-normal (LogN) fading and impulse noise conditions. In a recent work [12], multihop transmission is used for the low-frequency narrowband PLC network to resist the distance attenuation characteristics of power line channels. In these line channels, cooperative communication can reasonably select the relay nodes and use maximum ratio combining (MRC) or selective merging at the destination nodes to improve system reliability and ensure that normal communication can improve system performance even without a direct link. Reference [13] studies the dual-hop relay forwarding model of the power line based on an energy collection device, wherein the relay node can effectively improve the energy efficiency of the power line relay system via energy harvesting and effective power allocation strategy. In terms of wireless and PLC cooperation technologies, a system structure of parallel communication of wireless and power line is proposed in [14] and the bit error rate (BER) performance is analyzed under impulse noise. However, the fading coefficient of the power line is set to a fixed constant. Reference [15] adopts the indoor wireless and power line channel model of LogN to study the reliability of an indoor dual-media cooperative communication system based on AF and DF protocols and analyze the rules of LogN fading and power affecting system performance. In this study, the Gauss and Middleton-A noise models are used to model the two transmission media, and the fading models are all LogN distributions. The closed expression of the system performance can be obtained by using an approximate algorithm. Mathur et al. [16] analyze the average BER performance of the power line/wireless hybrid cooperative communication system when using dual-hop parallel communication and decoding and forwarding protocols. Similarly, reference [17] verifies the complementarity between PLC and wireless communication through measured data and obtains significant diversity gain by using MRC and selective combining. These two studies on hybrid fading systems are both focused on the DF protocol, hard decisions can result in the loss of useful information and error code, and its performance is significantly worse than that of the AF protocol. Study [18] also proposes a hybrid architecture of wireless and PLC networks installed in rural and sparsely populated environments to provide broadband and smart grid services.

Most of the works in the literature are focused on the performance of a dual-hop wireless or PLC system. In such a system, performance analysis is simplified owing to the symmetry of the system model as both links are subjected to the same channel and noise. Compared with existing investigations, the present study uses the hybrid fading model of wireless Nakagami-m/power line LogN and power line impulse noise to analyze the system performance of the AF relay protocol for the dual-media cooperative communication system of wireless access and power line relay. A good research method has not been put forward in existing literature to analyze this type of complicated and non-intuitive system. The proposed model is different from the symmetrical dual-hop system considered in the literature and is also a scientific problem to be developed that can provide significant theoretical basis for relevant research. The main contributions of this study are as follows:

(1) System performance is analyzed based on the mutual approximation of different channel fading. Considering the similarity between LogN and Gamma distributions, we propose an *s* vectors joint optimization algorithm based on moment generating function (MGF) equations and the principle of minimum difference between PDFs to determine the key parameters of approximate fading PDF and achieve high approximate accuracy.
(2) After the LogN distribution is approximated to the Gamma distribution (L2G), we derive the MGF of the harmonic mean of two Gamma distribution variables with the Appell function F1 form and use the MGF to obtain the BER, outage probability, and diversity order of the system. Compared with other studies, the MGF derived in this study must only calculate the Appell hypergeometric function once. This function has high accuracy, low complexity, and is suitable for any Nakagami-m fading parameter value.
(3) This study proposes an algorithm similar to (1) for determining the optimal distribution parameters and optimal *s* values based on the approximation of the LogN variable sum. The Gamma distribution is approximated to the LogN distribution (G2L) based on the principle of minimizing the MGF difference. In addition, the Gauss-Hermite series, integral changes, Mehta, and other algorithms are comprehensively applied to analyze the performance indicators of the double LogN distribution system.
(4) The versatility and accuracy of the two approximation algorithms are verified by using the Monte Carlo simulation, and the difference of the L2G and G2L algorithms is compared and analyzed in the calculated system performance for effectiveness and reliability. At the same time, the influence of the hybrid channel fading and multidimensional impulse noise on the system performance is analyzed. The simulation results show that both approximation algorithms have high accuracy, but the L2G algorithm can analyze system performance more accurately, and the G2L algorithm can be used to analyze other performances besides BER and outage since the PDF of terminal signal to noise ratio (SNR) is known.

The rest of the paper is organized as follows. Section 2 discusses the wireless/power line hybrid fading system model of the AF relay protocol. Section 3 calculates the average BER and outage probability based on the proposed approximation algorithm to evaluate the performance of the system under consideration. Section 4 presents the analysis and simulation results. Finally, Section 5 draws the conclusions.

## 2. System Description

**Figure 1:**
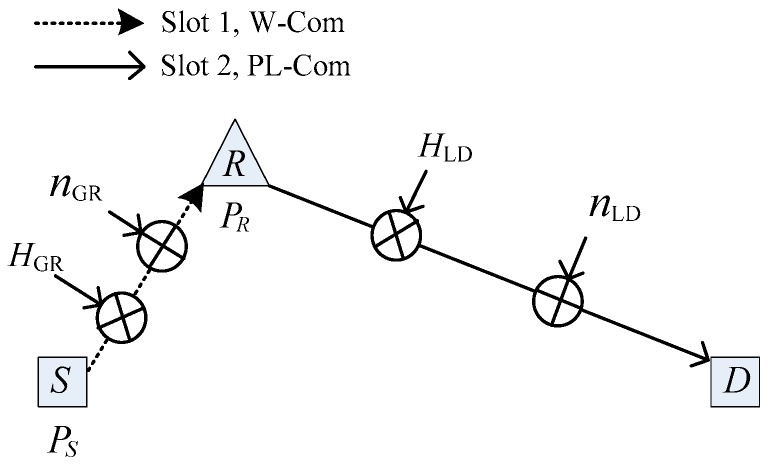
System model for a dual-hop wireless/power line hybrid communication system with amplify-and-forward (AF) relay.

Figure 1 shows the two-slot dual-media hybrid communication system considered in this work. In the first time slot, the source (*S*) uses the transmit power *P*_S_ to communicate wirelessly with the relay (*R*). In the second time slot, *R* amplifies and forwards the received data and transmits these to the terminal (*D*) through the power line by power *P*_R_.

The source *S* in Figure 1 refers to power equipment or independent smart meters or sensors, such as smart meters in buildings and homes, wireless sensor nodes in underground substations, non-contact infrared temperature cameras in substations, mobile Radio Frequency Identification card readers, etc. This system model combines the advantages of wireless and PLC technologies to provide consumers with the last kilometer access scheme, thereby effectively solving the distance limitation problem of the traditional wireless communication system. It likewise provides the infrastructure for many old buildings and residences that cannot achieve smart home conditions and effectively reduces cost. This system can also connect to the Internet in any room with weak wireless signals and expand network coverage. The system model uses mobile access and dual-hop relay technologies, which meet the practical requirements of flexible access and long-distance reliable transmission in smart grid communication.

### 2.1. First Time Slot

In the first time slot, *S* transmits wireless information to *R* by transmitting power *P*_S_ and *R* receives the following signals:

(1) $$y_{\mathrm{GR}}=H_{\mathrm{GR}}\sqrt{P_{S}}X_{S}+n_{\mathrm{GR}},$$

where the noise *n*_GR_ satisfies the normal distribution *N(0,N*_W_*)*, and *H*_GR_ is the wireless fading coefficient that satisfies the following Nakagami-m distribution.

(2) $$f_{}(H_{\mathrm{GR}}^{};m_{R},\Omega_{R})=\frac{2}{\Gamma (m_{R})}(\frac{m_{R}}{\Omega_{R}})m_{R}H_{\mathrm{GR}}^{2m_{R}-1}\exp(-\frac{m_{R}{\left|H_{\mathrm{GR}}^{}\right|}^{2}}{\Omega_{R}}),$$

where *m*_R_ ≥ 0.5 is the Nakagami-m fading parameter, Γ(x) is the gamma function, and Ω_R_ = E($|H_{\mathrm{GR}}{|}^{2}$) is the variance of the fading amplitude that is normalized to ensure that the fading does not change the average power of the received signal. Let Ω_R_ = 1.

According to the system model, let Δ_GR_ = *P*_S_*/N*_W_ denote the channel average SNR. Then, according to Equation (1), the instantaneous SNR $\gamma_{\mathrm{GR}}$ of the wireless channel *R* can be obtained as follows:

(3) $$\gamma_{\mathrm{GR}}=H_{\mathrm{GR}}^{2}\Delta_{\mathrm{GR}}=\frac{P_{S}|H_{\mathrm{GR}}^{}{|}^{2}}{N_{W}}.$$

Let $H_{2—,\mathrm{GI}}^{}={\left|H_{\mathrm{GI}}^{}\right|}^{2}$, (I=R,D), $H_{2—,\mathrm{GR}}^{}$ satisfies the Gamma distribution and is defined as follows:

(4) $$G(H_{2—,\mathrm{GR}}^{};\alpha_{R},\beta_{R})=\frac{{\left(H_{2—,\mathrm{GR}}^{}\right)}^{\alpha_{R}-1}}{\beta_{R}^{\alpha_{R}}\Gamma (\alpha_{R})}\exp(-\frac{H_{2—,\mathrm{GR}}^{}}{\beta_{R}}),$$

where the parameter relationship between the two distributions of Gamma and Nakagami-m satisfies *α*_R_ = *m*_R_, *β*_R_ = Ω_R_/*m*_R_.

Based on the properties of the Gamma function under the same average SNR, when Δ_GR_ is a constant, ${\left|H_{\mathrm{GR}}^{}\right|}^{2}\Delta_{\mathrm{GR}}\sim G(\gamma_{\mathrm{GR}};m_{R},\Delta_{\mathrm{GR}}\Omega_{R}/m_{R})$.

### 2.2. Second Time Slot

In the second time slot, *R* uses the AF protocol to forward the received data to *D* by power *P*_R_. Let *X*_R_ be the signal of *R*, then the signal $y_{\mathrm{LD}}$ received by *D* is

(5) $$y_{\mathrm{LD}}=H_{\mathrm{LD}}\sqrt{P_{R}}X_{R}+n_{\mathrm{LD}},$$

where $H_{\mathrm{LD}}$ is the power line fading coefficient that satisfies the following LogN($\mu_{D}^{},\sigma_{D}^{2}$) distribution:

(6) $$F_{}(H_{\mathrm{LD}};\mu_{D}^{},\sigma_{D})=\frac{1}{H_{\mathrm{LD}}\sigma_{D}\sqrt{2\pi }}\exp(-\frac{{\left(\ln H_{\mathrm{LD}}-\mu_{D}^{}\right)}^{2}}{2}),$$

where $\mu_{D}^{}$ and $\sigma_{D}$ are the mean and mean square deviation of $\ln H_{\mathrm{LD}}$, respectively. Let E(${\left|H_{\mathrm{LD}}^{}\right|}^{2}$) = exp(2*µ*_D_ + 2*σ*_D_^2^) = 1, that is, *µ*_D_ = −*σ*_D_^2^. The envelope energy of the channel fading can be normalized to ensure that the channel fading does not change the average power of the signal. On the basis of the properties of the LogN distribution, ${\left|H_{\mathrm{LD}}^{}\right|}^{2}$ satisfies the LogN($2\mu_{D}^{},4\sigma_{D}^{2}$) distribution.

To accurately characterize the PLC channel, the noise is assumed to consist of background and impulsive noise components [19]. These noise types are modeled using the two-term Bernoulli-Gaussian noise model [20]. Therefore, the PDF of the total noise can be simply expressed as

(7) $$n_{\mathrm{LD}}=n_{g}+n_{i}^{},$$

where $n_{g}$ is considered the Gaussian with zero mean and variance $\sigma_{g}^{2}$, while the impulsive part $n_{i}$ is modeled as the Bernoulli-Gaussian random process. As $n_{\mathrm{LD}}$ is the total noise $n_{i}=bA$, *A* is the white Gaussian noise with mean zero, and *b* is the Bernoulli process with the probability mass function.

(8) $$\begin{array}{l} P_{r}(b=1)=p\\ P_{r}(b=0)=1-p \end{array}$$

Therefore, the PDF of the total noise can be simply expressed as

(9) $$f_{n_{\mathrm{LD}}}=(1-p)N(0,\sigma_{g}^{2})+pN(0,\sigma_{i}^{2}+\sigma_{g}^{2}),$$

where $N(0,\sigma_{}^{2})$ refers to the Gaussian distribution with mean zero and $\sigma_{}^{2}$ as variance, and *p* is the arrival probability of the impulsive component of the Bernoulli-Gaussian noise. The noise power on the PLC link is $\sigma_{g}^{2}$ in the presence of only the background noise, while the total noise power is $\sigma_{g}^{2}(1+T)$ in the presence of background and impulsive noises, where $T=\sigma_{i}^{2}/\sigma_{g}^{2}$ is the impulsive noise index.

Let Δ_LD_ *= P*_R_/*N*_P_, and then the instantaneous SNR of the power line receiver can be expressed as

(10) $$\gamma_{\mathrm{LD}}={\left|H_{\mathrm{LD}}^{}\right|}^{2}\Delta_{\mathrm{LD}}.$$

Therefore, the total SNR $\gamma_{\mathrm{GL}}^{}$ of the dual-hop AF relay protocol communication system is

(11) $$\gamma_{\mathrm{GL}}^{}=\frac{\gamma_{\mathrm{GR}}\gamma_{\mathrm{LD}}}{\gamma_{\mathrm{GR}}+\gamma_{\mathrm{LD}}+1}.$$

When a high SNR (*P*_S_*/N*_W_, *P*_R_/*N*_P_≫1) exists, Equation (11) can be approximated as

(12) $$\gamma_{\mathrm{GL}}^{}\approx \frac{\gamma_{\mathrm{GR}}\gamma_{\mathrm{LD}}}{\gamma_{\mathrm{GR}}+\gamma_{\mathrm{LD}}}=\frac{1}{1/\gamma_{\mathrm{GR}}+1/\gamma_{\mathrm{LD}}}.$$

## 3. Performance Analysis

### 3.1. Mutual Approximation Algorithm Based on the PDF and MGF Equations

Determining the PDF of each branch SNR is necessary to obtain the system’s outage probability and BER. However, the PDF of the destination node *D* of the hybrid fading system is difficult to solve, making it difficult, in turn, to analyze the system performance. At the same time, it is mathematically difficult to deal with because the LogN variable integration does not have a closed expressionin the process of analyzing the performance of the system BER. Therefore, the ${\left|H_{\mathrm{LD}}^{}\right|}^{2}$ of LogN($2\mu_{D}^{},4\sigma_{D}^{2}$) distribution is approximated to $G(H_{2—,\mathrm{GD}}^{};\alpha_{D},\beta_{D})$ based on the similarity between two distributions [21] for further analysis of the system.

The approximation accuracy is poor because the PDF approximation is obtained in the same way as the mean and variance [22]. Therefore, we propose the PDF approximation algorithm based on the MGF equations in this work, and the obtained approximation function has high precision.

${\left|H_{\mathrm{LD}}^{}\right|}^{2}$ satisfies the LogN($2\mu_{D}^{},4\sigma_{D}^{2}$) distribution, and the MGF is presented as

(13) $$M_{\mathrm{LD}}(s;\mu_{D},\sigma_{D})=\sum_{n=1}^{N}\frac{w_{n}}{\sqrt{\pi }}\exp(-s_{i}\exp(\sqrt{2}2\sigma_{D}a_{n}+2\mu_{D})),$$

where $s_{i}{}^{{}^{}}(i=1,2)$ is an adjustable variable. *N* is the Hermite integration order, and $w_{n}$ and $a_{n}$ are the weights of the Gauss-Hermite formula and their abscissas [23], respectively.

Similarly, the MGF equation of $G(H_{2—,\mathrm{GD}}^{};\alpha_{D},\beta_{D})$ is satisfied with the following equation:

(14) $$M_{\mathrm{GD}}((s;\alpha_{D},\beta_{D}))={\left(1+\beta_{D}s_{i}\right)}^{-\alpha_{D}}.$$

Then the L2G of ${\left|H_{\mathrm{LD}}^{}\right|}^{2}$ using the MGF equations $M_{\mathrm{GD}}((s;\alpha_{D},\beta_{D})=M_{\mathrm{LD}}(s;\mu_{D},\sigma_{D})$. The approximated parameters *α*_D_ and *β*_D_ can be solved by using the fsolve function in MATLAB.

(15) $$\sum_{n=1}^{N}\frac{w_{n}}{\sqrt{\pi }}\exp(-s_{i}\exp(\sqrt{2}2\sigma_{D}a_{n}+2\mu_{D})={\left(1+\beta_{D}s_{i}\right)}^{-\alpha_{D}}.$$

The values of the independent variables *s*_1_ and *s*_2_ in the MGF equations are directly related to the determination of the approximate parameters *α*_D_ and *β*_D_ and the approximate accuracy of the PDF. This study proposes a mathematical optimization model based on the *s* vectors optimization algorithm with the minimum difference of the PDF curves, the key parameters of the approximate fading PDF are obtained by solving a set of optimal *s*_1_ and *s*_2_ combinations. For the known power line fading parameters $\mu_{D}^{}$ and $\sigma_{D}$, we use PDF Equations (4) and (6) to solve the wireless fading parameters $\alpha_{D}$ and $\beta_{D}$ with *s*_1_ and *s*_2_ as variables. Hence, the following mathematical model is established:

(16) $$\underset{s1,s2}{\min}\sum_{i=1}^{N}{\left(F(H_{i};2\mu_{D},2\sigma_{D})-G(H_{i};\alpha_{D},\beta_{D})\right)}^{2},$$

subject to:

(1) $\sum_{n=1}^{N}\frac{w_{n}}{\sqrt{\pi }}\exp(-s_{1}\exp(\sqrt{2}2\sigma_{D}a_{n}+2\mu_{D}))={\left(1+\beta_{D}s_{1}\right)}^{-\alpha_{D}}$;
(2) $\sum_{n=1}^{N}\frac{w_{n}}{\sqrt{\pi }}\exp(-s_{2}\exp(\sqrt{2}2\sigma_{D}a_{n}+2\mu_{D}))={\left(1+\beta_{D}s_{2}\right)}^{-\alpha_{D}}$;
(3) *H_i_* = 0.01 + 0.05 × (*i* − 1);
(4) *i* = 1,2, …, *N*;
(5) *s*_1_ > 0;
(6) *s*_2_ > 0;

where *H_i_* denote the sample values of the PDF of SNR with fading, and *N* is the total number of sample values of the PDF (*N* = 100 is selected in this study).

The mathematical model considers the matching degree of PDF curves of the LogN and Gamma distributions as the optimization objective and performs the weighting calculation on the square of the difference of the PDF values to the corresponding fading sampling values *H_i_*. Specifically, $\sum_{i=1}^{N}{\left(F(H_{i},2\mu_{D},2\sigma_{D})-G(H_{i},\alpha_{D},\beta_{D})\right)}^{2}$, which is called the objective function in this study. When the selected *s* value minimizes the objective function, the simultaneous equations can obtain the PDF key parameters of approximated Gamma distribution by the known LogN distribution. The optimal *s* value can be solved by an intelligent optimization algorithm, such as the genetic algorithm.

**Figure 2:**
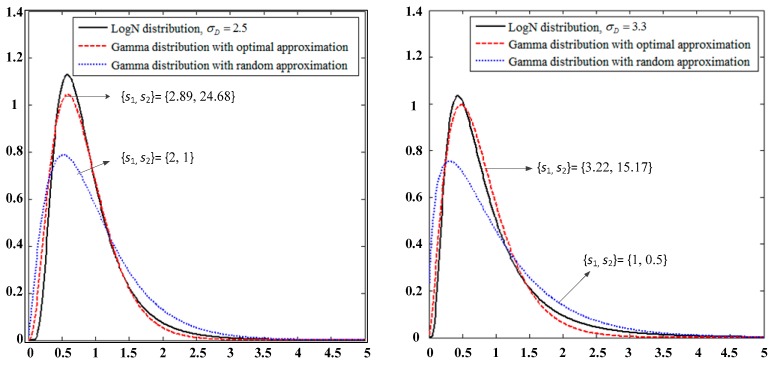
Probability density function (PDF) comparison of ${\left|H_{\mathrm{GD}}^{}\right|}^{2}$ with the Log-normal (LogN) and approximated Gamma distributions.

Therefore, the numerical calculation and analysis are conducted in this study under channel fading normalization (E(${\left|H_{\mathrm{LD}}^{}\right|}^{2}$) = 1). Figure 2 presents the PDF curves comparison of the LogN distribution in the second time slot of the system with the approximated Gamma distribution for the different combinations of parameters *s*_1_, *s*_2_ and power line fading parameter $\sigma_{D}^{}$.

(1) (The choice of *s* value that will affect the prediction of the PDF curve coincidence is verified.
(2) Although the MGF equation approximation lacks analytical expressions, it can be optimized through *s*_1_ and *s*_2_ with increased accuracy.

**Figure 3:**
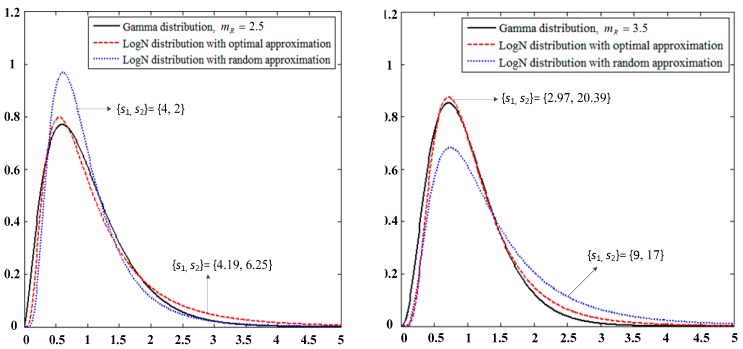
PDF comparison of ${\left|H_{\mathrm{LR}}^{}\right|}^{2}$ with the Gamma and approximated LogN distributions.

For the sake of clarity, Figure 3 presents the PDF curve comparison of the Gamma distribution in the first time slot with the approximated LogN distribution under normalized conditions (E(${\left|H_{\mathrm{GR}}^{}\right|}^{2}$) = 1) for different combinations of the parameters *s*_1_, *s*_2_,and *m*_R_.

Using the above method, Table 1 presents the approximate optimization table of the MGF equation for reference in practical applications, where *s*_1_ and *s*_2_ are the optimal values of different *σ*_D_, and *m*_D_ and β_D_ are the PDF parameters that are optimal approximated to the Gamma distribution by the usual *σ*_D_, *s*_1_ and *s*_2_.

Similarly, Table 2 shows the optimization results of the LogN distribution approximated by the parameters of the Gamma distribution of the first time slot in the system.

As the fading parameters of the channel after the approximation no longer satisfy the normalization condition, it would be cumbersome to use above numerical experimental method to determine the appropriate s_1_ and s_2_ values. In addition, the accuracy of s_1_ and s_2_ determined by the experiments in a large numerical range will not be guaranteed because of the lack of help from mathematical theory tools. Thus, this study first approximates the normalized PDF of channel fading and then calculates the equivalent parameters of the instantaneous SNR based on the average SNR of the first hop. According to $\gamma_{\mathrm{LD}}^{}=H_{\mathrm{LD}}^{2}\Delta_{\mathrm{LD}}$ and its properties, the key parameters of $\gamma_{\mathrm{LD}}^{}$ approximating into $G(\gamma_{\mathrm{GD}}^{};\bar{\alpha_{D}},\bar{\beta_{D}})$ distribution are

(17) $$\bar{\alpha_{D}}=\alpha_{D}^{},$$

(18) $$\bar{\beta_{D}}=\beta_{D}^{}\cdot \Delta_{\mathrm{LD}}.$$

**Figure 4:**
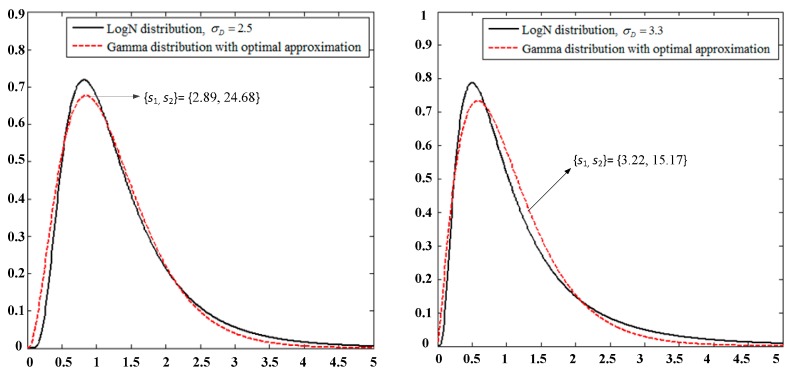
Comparison of PDF for instantaneous SNR with the LogN and approximated Gamma distributions using the best *s* value optimization method.

Figure 4 shows a comparison of PDF for L2G based on the joint optimization algorithm of MGF equations and *s* vectors when Δ=2.

**Figure 5:**
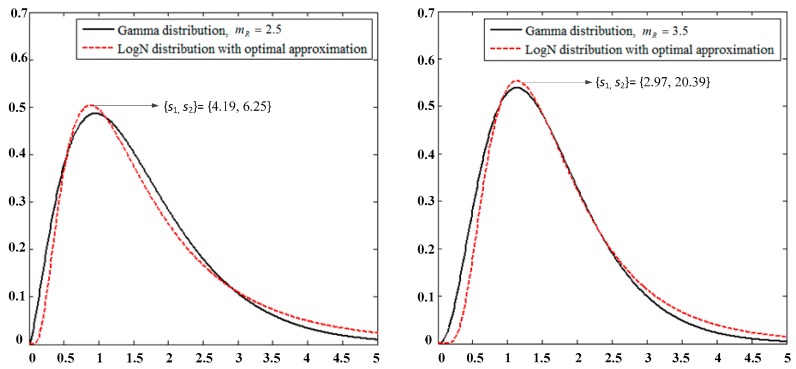
Comparison of PDF for instantaneous SNR with the Gamma and approximated LogN distributions using the best *s* value optimization method.

Similarly, in the approximation process of the Gamma distribution to the LogN distribution of Figure 5, according to $\gamma_{\mathrm{GR}}^{}=H_{\mathrm{GR}}^{2}\Delta_{\mathrm{GR}}$, the key parameters after the variable $\gamma_{\mathrm{GR}}^{}$ is approximated to LogN($\bar{\mu_{R}},{\bar{\sigma_{R}^{2}}}^{\prime }$) are $\bar{\sigma_{R}^{2}}=\sigma_{R}^{2}$ and $\bar{\mu_{R}}=\ln\Delta_{\mathrm{GR}}+\mu_{R}^{}$.

### 3.2. Performance Analysis of MGF Based on Harmonic Mean of the Double Gamma Distribution System

The performance analysis under hybrid fading is transformed into the performance analysis under the double Gamma distribution after L2G in the second time slot *R*–*D* link. Many studies have investigated the BER and outage performance of the AF relaying system under Nakagami-m fading. However, these studies have the following limitations: (1) The use of the approximate function for end-to-end SNR, such as γ_GR_γ_GD_/(γ_GR_ + γ_GD_ + 1) ≈ min(γ_GR_,γ_GD_) [24], leads to imprecise results. (2) Numerous performance formulas contain multiple series [25], Whittaker functions [26], or Appell F4 functions [27], thereby causing high computation complexity. (3) Some methods can only evaluate the system outage probability [28]. (4) Some precise conclusions are only suitable for a special fading parameter m (integer [24,25] or integer plus one-half [25]). To reduce the computational complexity of the harmonic mean, some studies use the least function approximation calculation. Some basic frame works are also proposed but with increased complexity, where in two Appell functions need to be calculated [16]. Motivated by all of the above, we present an effective performance evaluation approach using the basic theory of probability. The MGF for terminal-to-terminal instantaneous SNR is derived first in the form of the Appell function F1, which involves a single integral that can be computed by the general software MATLAB. Suitable for the arbitrary Nakagami-m fading parameter m, the MGF is then used to derive the system performance of the diversity order, BER, and outage probability.

The channel coefficients *H*_GR_ and *H*_GD_ of the *S–R* and *R*–*D* links are subject to Nakagami-m fading with the shape parameters *m*_R_ and *m*_D_ and satisfy E[|*H*_GR_|^2^] = Ω_R_ = 1 and E[|*H*_GD_|^2^] = *Ω*_D_. Then, the instantaneous SNR of the links are γ_GR_ = |*H*_GR_|^2^Δ_GR_ and γ_GD_ = |*H*_GD_|^2^Δ_GD_, where Δ_GR_ and Δ_GD_ are the average SNRs. Consequently, each γ_GI_(I = R, D) satisfies the Gamma distribution denoted as γ_GI_~*G*(γ_GI_; *α*_I_, *β*_I_), where *α*_I_ = *m*_I_ and *β*_I_ *=* Δ_GI_*Ω*_I_/*m*_I_. The equivalent end-to-end SNR γ_GG_ at terminal *D* is similar to that of Equation (11). *F*_1_(·) represents the first type of Appell hypergeometric function, and the MGF of the harmonic mean of the two variables with the Gamma distribution satisfies the following lemma.

**Lemma** **1.** *If X_I_(I = R,D) satisfies G(X_I;_ α_I_, β_I_), then the MGF of X_I_’s harmonic mean Z = X_R_*×*X_D_/(X_R_ + X_D_) has the following expression:*

(19) $$M_{Z}(s;\alpha_{R},\alpha_{D},\beta_{R},\beta_{D})={\left(\frac{\beta_{D}}{\beta_{R}}\right)}^{\alpha_{D}}\times F_{1}\left(\alpha_{D};\alpha_{R}+\alpha_{D},\alpha_{R}+\alpha_{D};\alpha_{R}+\alpha_{D};k_{1},k_{2}\right),$$

*where*$k_{1}=\beta_{R}\beta_{D}s/\left(\rho -\sqrt{\beta_{R}\beta_{D}^{2}s+\rho^{2}}\right)$*,*$k_{2}=\beta_{R}\beta_{D}s/\left(\rho +\sqrt{\beta_{R}\beta_{D}^{2}s+\rho^{2}}\right)$*, and*$\rho =(\beta_{R}\beta_{D}s+\beta_{R}-\beta_{D})/2$.

**Proof.**  We mainly derive the PDF and MGF for γ_GI_. Then, by using the PDF of γ_GI_, which is denoted as $f_{\gamma_{\mathrm{GI}}}(x)$, we can obtain the PDF of 1/$\gamma_{\mathrm{GI}}$, *Y* = 1/$\gamma_{\mathrm{GR}}$ *+* 1/$\gamma_{\mathrm{GD}}$ and *Z* = 1/*Y*, which are presented as follows:

(20-a) $$f_{1/\gamma_{\mathrm{GI}}}(x)=\frac{1}{x^{2}}f_{\gamma_{\mathrm{GI}}}\left(\frac{1}{x}\right)\cdot U(x),$$

(20-b) $$f_{Y}(y)=\int_{0}^{y}f_{1/\gamma_{\mathrm{GR}}}(y-x)f_{1/\gamma_{\mathrm{GD}}}(x)dx\cdot U(y),$$

(20-c) $$\begin{array}{l} f_{Z}(z)=\frac{1}{z^{2}}\int_{0}^{\frac{1}{z}}f_{1/\gamma_{\mathrm{GR}}}\left(\frac{1}{z}-x\right)f_{1/\gamma_{\mathrm{GD}}}\left(x\right)dx\cdot U(z)\\ \underline{\underline{x=t/z}}\overset{}{}z\int_{0}^{1}\frac{1}{t^{2}{\left(1-t\right)}^{2}}f_{\gamma_{\mathrm{GR}}}\left(\frac{z}{1-t}\right)f_{\gamma_{\mathrm{GD}}}\left(\frac{z}{t}\right)dt\cdot U(z) \end{array}$$

where *U*(*z*) = 1 for *z* ≥ 0 and *U*(*z*) = 0 for *z* < 0. Then, by substituting the PDF of $\gamma_{\mathrm{GI}}$, which is *G*(*X*_I;_ *α*_I_, *β*_I_) into Equation (20-c), we derive the following:

(21) $$f_{Z}(z)=\int_{0}^{1}\frac{z^{\alpha_{R}+\alpha_{D}-1}\exp\left(-z\left(\frac{1}{\beta_{R}t}+\frac{1}{\beta_{D}(1-t)}\right)\right)}{\Gamma \left(\alpha_{R}\right)\Gamma \left(\alpha_{D}\right)\beta_{R}^{\alpha_{R}}\beta_{D}^{\alpha_{D}}t^{\alpha_{R}+1}{\left(1-t\right)}^{\alpha_{D}+1}}dt\cdot U(z).$$

According to the MGF definition $M_{Z}\left(s\right)=\int_{0}^{\infty }e^{-sz}f_{Z}(z)dz$, the following expression is obtained:

(22) $$\begin{array}{l} M_{Z}(s;\alpha_{R},\alpha_{D},\beta_{R},\beta_{D})=\int_{0}^{1}\frac{1}{\Gamma \left(\alpha_{R}\right)\Gamma \left(\alpha_{D}\right)\beta_{R}^{\alpha_{R}}\beta_{D}^{\alpha_{D}}t^{\alpha_{R}+1}{\left(1-t\right)}^{\alpha_{D}+1}}\times \\ \left(\int_{0}^{\infty }z^{\alpha_{R}+\alpha_{D}-1}\exp\left(-\left(\frac{1}{\beta_{R}t}+\frac{1}{\beta_{D}(1-t)}+s\right)z\right)dz\right)dt \end{array}$$

When $\int_{0}^{\infty }x^{\nu -1}\exp(-ux)dx=\Gamma (v)/u^{v}$ (*u* > 0, *v* > 0) is used [23], the MGF of Equation (22) can be rewritten as

(23) $$M_{Z}(s;\alpha_{R},\alpha_{D},\beta_{R},\beta_{D})=\int_{0}^{1}\frac{\beta_{R}^{\alpha_{D}}\cdot \beta_{D}^{\alpha_{R}}\cdot t^{\alpha_{D}-1}{\left(1-t\right)}^{\alpha_{R}-1}}{B(\alpha_{R},\alpha_{D})\cdot {\left(\beta_{D}^{}+2\Delta t-\beta_{R}^{}\beta_{D}^{}st^{2}\right)}^{\alpha_{R}+\alpha_{D}}}dt=\int_{0}^{1}\Omega (s,t)dt,$$

where B(*α*_R_, *α*_D_) is the beta function. Finally, according to the definition of the Appell function F1 [23], a concise form of the MGF is obtained as

(24) $$M_{Z}(s;\alpha_{R},\alpha_{D},\beta_{R},\beta_{D})={\left(\frac{\beta_{D}}{\beta_{R}}\right)}^{\alpha_{D}}\times F_{1}\left(\alpha_{D};\alpha_{R}+\alpha_{D},\alpha_{R}+\alpha_{D};\alpha_{R}+\alpha_{D};k_{1},k_{2}\right).$$

Hence, the final MGF only involves a single integral, which can be solved by quad and other integral functions in MATLAB or Appell F1 function in Mathematics.

In particular, when the PDF parameters of *X*_R_ and *X*_D_ are the same, that is, $\alpha_{R}=\alpha_{D}=\alpha$ and $\beta_{R}=\beta_{D}=\beta$, Equation (19) can be defined as

(25) $$M_{Z}(s;\alpha ,\beta )=\int_{0}^{1}\frac{\Gamma (2\alpha_{})\beta^{2\alpha }}{\Gamma (\alpha_{})\Gamma (\alpha_{})}\frac{{\left[t(1-t)\right]}^{\alpha -1}}{{\left[\beta +\beta^{2}s\cdot t(1-t)\right]}^{2\alpha }}dt.$$

Finally, through integral transformation, the above equationcan be simplified to

(26) $$M_{Z}(s;\alpha ,\beta )={}_{2}F{}_{1}\left(2\alpha ,\alpha ;\alpha +\frac{1}{2};-\frac{\beta }{4}s\right),$$

where ${}_{2}F{}_{1}(\cdot )$ represents the hypergeometric function [29]. The function ${}_{2}F{}_{1}(\cdot )$ can also be expressed as the following simple integral:

(27) $${}_{2}F{}_{1}\left(2\alpha ,\alpha ;\alpha +\frac{1}{2};-\frac{\beta }{4}s\right)=\frac{\Gamma (\alpha +\frac{1}{2})}{\Gamma (\alpha )\Gamma (\frac{1}{2})}\int_{0}^{1}\frac{t^{\alpha -1}{\left(1-t\right)}^{-\frac{1}{2}}}{{\left(1-tz\right)}^{2\alpha }}dt.$$

For the coherently *M*-PSK modulation, the system BER is defined as

(28) $$P_{\mathrm{BER}}^{\mathrm{GG}}=\frac{1}{\pi }\cdot \int_{0}^{\frac{(M-1)\pi }{M}}M_{Z}(\frac{g_{\mathrm{PSK}}}{\sin^{2}\theta };\alpha_{R},\alpha_{D},\beta_{R},\beta_{D})d\theta .$$

With the help of the MGF derived in Equation (22), we can then compute the BER according to [27] or use the double integral function in MATLAB directly, where *g*_PSK_ = sin^2^(π/*M*). If $\Theta =\frac{(M-1)\pi }{M}$, Equation (28) can be further simplified to

(29) $$\begin{array}{l} P_{\mathrm{BER}}^{\mathrm{GG}}\approx \left(\frac{\Theta }{2\pi }-\frac{1}{6}\right)M_{Z}\left(g_{\mathrm{PSK}};\alpha_{R},\alpha_{D},\beta_{R},\beta_{D}\right)+\frac{1}{4}M_{Z}\left(\frac{4}{3}g_{\mathrm{PSK}};\alpha_{R},\alpha_{D},\beta_{R},\beta_{D}\right)\\ +\left(\frac{\Theta }{2\pi }-\frac{1}{4}\right)M_{Z}\left(\frac{g_{\mathrm{PSK}}}{\sin^{2}\Theta };\alpha_{R},\alpha_{D},\beta_{R},\beta_{D}\right) \end{array}$$

Similarly, the outage probability *P*_out_ is defined as the probability value when the instantaneous total SNR is lower than the fixed threshold (*R*_th_). The outage probability can also be calculated by substituting Equation (22) into the following equation [25] after some necessary alterations:

(30) PoutGG=P(Rth;A,N,Q)=2−QeA/2Rth∑q=0Q(Qq)⋅∑n=0N+q(−1)nβnRe{MZ(A+2πjn2Rth;αR,αD,βR,βD)A+2πjn2Rth}+E(A,N,Q)

where *β*_0_ = 2 and *β*_n_ = 1(*n* is a positive integer), and the values of *A*, *N* and *Q* determine the accuracy of the numerical calculation, which is estimated by the overall truncation error term *E*(*A*, *N*, *Q*). □

**Lemma** **2.** *If X_I_(I = R,D) satisfies G(X_I;_ α_I_, β_I_), the diversity order of the AF relay system is min(α_R_, α_D_). See Appendix A for the specific certification process.*

### 3.3. Performance Analysis of the Double LogN Distribution System Based on the Approximation of the LogN Variable Sum

In this section, we will similarly operate G2L in the first time slot of the system to analyze the performance. In particular, the system performance analysis under the hybrid fading condition is converted into the double LogN distribution condition. Accordingly, the best approximation scheme can be further selected by comparing the accuracy, reliability, and complexity of the two approximation algorithms with those in Sub-Section 3.2. In addition, the method directly calculates the BER and outage probability of the system by using the SNR of the system terminal *D*, which is beyond the limitation of the MGF and can easily analyze various performance indexes of the system.

We assume that the process of approximating the Nakagami-m distribution to the LogN distribution in the first slot has been completed because of the similarity of the approximation process (Section 3.1). Figure 3 shows the comparison of the PDF curves of the approximate ${\left|H_{\mathrm{LR}}^{}\right|}^{2}$. At this time, *H*_LR_ and *H*_LD_ are the respective power line fading coefficients of the first and second time slots of the system model, which satisfy the LogN($\mu_{I}^{},\sigma_{I}^{2}$) distribution, (I = R,D). The instantaneous SNR of the link is γ_LI_ = |*H*_LI_|^2^Δ_LI_, where Δ_LR_ *= P*_S_/*N*_W_ and Δ_LD_ *= P*_R_/*N*_P_ are the average SNRs of the channel. Then, the total SNR γ_LL_ of the AF relay protocol communication system can be directly written as

(31) $$\gamma_{\mathrm{LL}}=\frac{1}{\frac{1}{P_{S}|H_{\mathrm{LR}}{|}^{2}/N_{W}}+\frac{1}{P_{R}|H_{\mathrm{LD}}{|}^{2}/N_{P}}}.$$

The instantaneous mutual information *I* of the system is

(32) $$I=\frac{1}{2}\log(1+\gamma_{\mathrm{LL}}^{}).$$

According to the properties of the LogN distribution, *P*_S_|*H*_LR_|^2^/*N*_W_ and *P*_R_|*H*_LD_|^2^/*N*_P_ satisfy the LogN distribution when *P*_S_/*N*_W_ and *P*_R_/*N*_P_ are constants. As the reciprocal of the LogN variable still satisfies the LogN distribution, 1/(*P*_S_| *H*_LR_|^2^/*N*_W_) and 1/(*P*_R_|*H*_LD_|^2^/*N*_P_) also satisfy the LogN distribution. Hence, the reciprocal of the sum of the LogN distribution variables, namely, the SNR $\gamma_{\mathrm{LL}}^{}$ of the system, also satisfies the LogN distribution.

If $G=1/(P_{S}|H_{\mathrm{LR}}{|}^{2}/N_{W})+1/(P_{R}|H_{\mathrm{LD}}{|}^{2}/N_{P})$, then $\gamma_{\mathrm{LL}}\sim \mathrm{Log}(\mu_{G}^{},\sigma_{G}^{2})$. When the known parameters $\mu_{I}^{}$, $\sigma_{I}^{2}$ and the Mehta algorithm are used, the distribution parameters *µ*_G_ and *σ*_G_ of variable *G* satisfy the following relations:

(33) $$\begin{array}{l} \sum_{n=1}^{N}\frac{w_{n}}{\sqrt{\pi }}\exp(-s_{i}\exp(\sqrt{2}\sigma_{G}a_{n}+\mu_{G}))=\\ \sum_{n=1}^{N}\frac{w_{n}}{\sqrt{\pi }}\exp(-s_{i}\exp(\sqrt{2}\sigma_{A}a_{n}+\mu_{A}))\cdot \sum_{n=1}^{N}\frac{w_{n}}{\sqrt{\pi }}\exp(-s_{i}\exp(\sqrt{2}\sigma_{B}a_{n}+\mu_{B})) \end{array}$$

where *w*_n_ and *a*_n_ refer to literature [22], $P_{S}|H_{\mathrm{LR}}{|}^{2}/N_{W}\sim \mathrm{LogN}(\mu_{A},\sigma_{A}^{2}\;)$, $P_{R}|H_{\mathrm{LD}}{|}^{2}/N_{P}\sim \mathrm{LogN}(\mu_{B},\sigma_{B}^{2}\;)$. The two equations in the simultaneous Equation (33) can solve *µ*_G_ and *σ*_G_ by using the fsolve function of MATLAB. The Mehta algorithm has high precision, but the parameters of *µ*_G_ and *σ*_G_ obtained by equation solution lacks analytical expression. 

Similar to the modeling method of Equation (16), joint parameter optimization based on the principle of minimum difference of the MGF curve is conducted to fit the LogN distribution parameters of variable *G* using an objective function

(34) $$\underset{s1,s2}{\min}\sum_{k=1}^{N}{\left(M_{\mathrm{GR}}((s_{k};\alpha_{R},\beta_{R}))-M_{\mathrm{LR}}(s_{k};\mu_{R},\sigma_{R})\right)}^{2}.$$

If the threshold is *R*_th_ and *γ* = exp(2*R*_th_)−1, then the $P_{\mathrm{out}}^{\mathrm{LL}}$ is

(35) $$P_{\mathrm{out}}^{\mathrm{LL}}=P_{r}(I<R_{\mathrm{th}})\approx P_{r}(\gamma_{\mathrm{LL}}^{}<\gamma ).$$

By substituting the cumulative distribution function $F(\lambda )=Q(-(\ln\gamma +\mu_{G}))/\sigma_{G})$ of $\gamma_{\mathrm{LL}}^{}$ into Equation (35), we obtain

(36) $$P_{\mathrm{out}}^{\mathrm{LL}}=\sum_{j=0}^{1}p_{j}Q(—-\frac{\ln\gamma +\mu_{\mathrm{Gj}}}{\sigma_{\mathrm{Gj}}}),$$

where $p_{0}=1-p$, and $p_{1}=p$, which represent the probability of whether impulse noise exists in the power line channel. $\mu_{\mathrm{Gj}}$ and $\sigma_{\mathrm{Gj}}$ are the mean and variance of the attenuation distribution for the Gauss channel and the channel with impulse noise (j=0,1), respectively.

The BER $P_{\mathrm{BER}}^{\mathrm{LL}}$ of the system is obtained by using F(λ) in the following equation:

(37) $$P_{\mathrm{BER}}^{\mathrm{LL}}=-\sum_{j=0}^{1}p_{j}\int_{0}^{\infty }\frac{dQ(\sqrt{\lambda })}{d\lambda }F(\lambda )d\lambda =\sum_{j=0}^{1}p_{j}\int_{0}^{\infty }\frac{1}{2\sqrt{2\pi \lambda }}\exp(-\frac{\lambda }{2})Q(-\frac{\ln\gamma +\mu_{\mathrm{Gj}}}{\sigma_{\mathrm{Gj}}})d\lambda .$$

To simplify the calculation, let $t=(-\mu_{\mathrm{Gj}}-\ln\lambda )/\sigma_{\mathrm{Gj}}$, the integral transformation of the above equation is defined as follows:

(38) $$P_{\mathrm{BER}}^{\mathrm{LL}}=\sum_{j=0}^{1}p_{j}\int_{-\infty }^{\infty }\frac{\sigma_{\mathrm{Gj}}}{2\sqrt{2\pi }}\exp(-\frac{-\mu_{\mathrm{Gj}}-t\sigma_{\mathrm{Gj}}}{2})\cdot \exp(-\exp(-\mu_{\mathrm{Gj}}-t\sigma_{\mathrm{Gj}}+\ln0.5))\cdot Q(t)\cdot (-\sigma_{\mathrm{Gj}}\exp(-\mu_{\mathrm{Gj}}-t\sigma_{\mathrm{Gj}}))dt.$$

Then, exp(−exp(x)) is approximated to the form of Gaussian function weighting, and the BER $P_{\mathrm{BER}}^{\mathrm{LL}}$ of the system is expressed as follows [11]:

(39) $$P_{\mathrm{BER}}^{\mathrm{LL}}=-\sum_{j=0}^{1}\sum_{k=1}^{4}p_{j}\frac{Z_{\mathrm{kj}}}{X_{\mathrm{kj}}}Q(\frac{Y_{\mathrm{kj}}}{\sqrt{1+X_{\mathrm{kj}}^{2}}}),$$

which includes

(40) $$\begin{array}{l} X_{\mathrm{kj}}=\sqrt{2\sigma_{\mathrm{Gj}}^{2}/R_{3k}^{2}}\\ Y_{\mathrm{kj}}=\frac{4\sigma_{\mathrm{Gj}}(\mu_{\mathrm{Gj}}+\ln0.5-R_{2k})-\mu_{\mathrm{Gj}}R_{3k}^{2}}{2X_{k}R_{3k}^{2}}\\ Z_{\mathrm{kj}}=0.5R_{1k}\sigma_{\mathrm{Gj}}\exp(\frac{-\mu_{\mathrm{Gj}}+Y_{k}^{2}}{2})\exp[-{\left(\frac{-\mu_{\mathrm{Gj}}+\ln0.5-R_{2k}}{R_{3k}}\right)}^{2}] \end{array}$$

## 4. Numerical Results and Discussion

In this section, Monte Carlo computer simulation experiments are performed using MATLAB software to verify the reliability and accuracy of the theoretical formulas and illustrate the effects of various system parameters on the BER and outage probability of the system under binary phase shift keying modulation. In all simulations, the simulation process uses the following default settings unless mentioned otherwise: (1) The total system power is 2, $P_{S}=P_{R}=1$. (2) To highlight the impact of channel fading and noise on the performance, we assume that the average SNR of the system channel is Δ, *N*_W_ = *N*_P_ = 1/Δ, that is, Δ_GR_ = Δ_LD_ = Δ. (3) The system threshold $R_{\mathrm{th}}=0.2$. (4) The Bernoulli-Gaussian noise parameter p=0.1, T=10.

**Figure 6:**
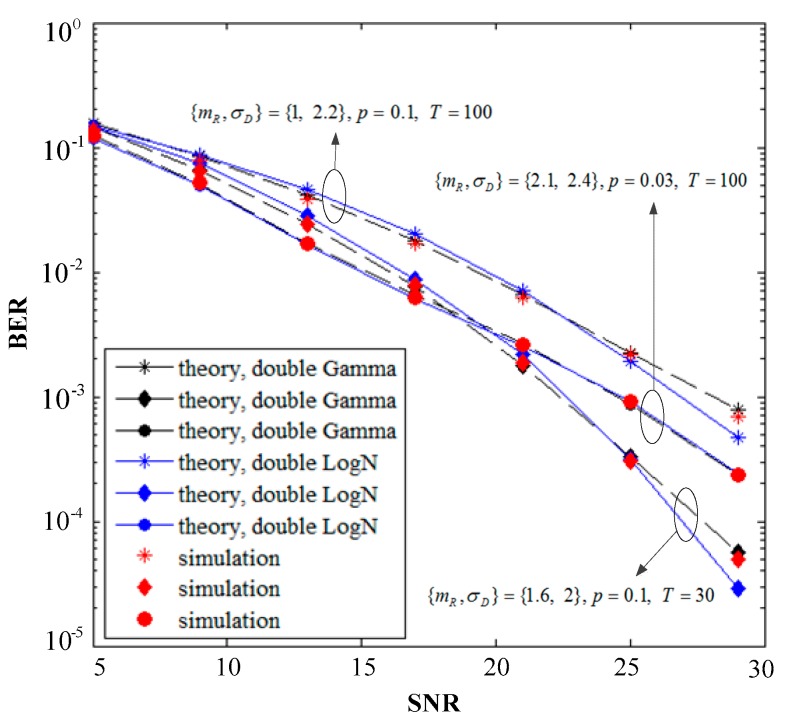
Comparison of analytical and simulated results of the system bit error rate (BER) and the per hop average SNR.

Figure 6 shows the BER performance of the simulated and theoretical calculations when the fading parameters are set to $\{m_{R},\sigma_{D}\}=\{1{,}^{}\;2.2\},\{{1.6,\;}^{}2\},\{{2.1,\;}^{}2.4\}$, the values of the Bernoulli-Gaussian noise parameters *p* and *T* are shown in the figure. The BER decreases as the system SNR increases, and the resulting curve matches well with the Monte Carlo simulations, thereby implying the correctness of the derived analytical expressions and the validity of the theoretical derivation of the two approximation algorithms. The coincidence degree between the theory and simulation of the L2G algorithm is likewise higher than that of the G2L algorithm in terms of algorithm comparison, especially at high SNR, which is caused by multiple approximations in the theoretical calculation of the latter algorithm. The L2G algorithm is highly accurate and suitable for arbitrary *m* values rather than special parameters, which are reflected in the BER curves of the three sets of parameters in the figure. However, the simulation process of the L2G algorithm is sensitive to the selection of parameters, and such sensitivity is related to the high complexity of the algorithm and the large proportion of integrals involved. Although the G2L algorithm is not accurate enough, the theoretical calculation is simply for end-to-end SNR and multiple approximations, which can be used to analyze many performance indicators and solve a wide range of problems.

**Figure 7:**
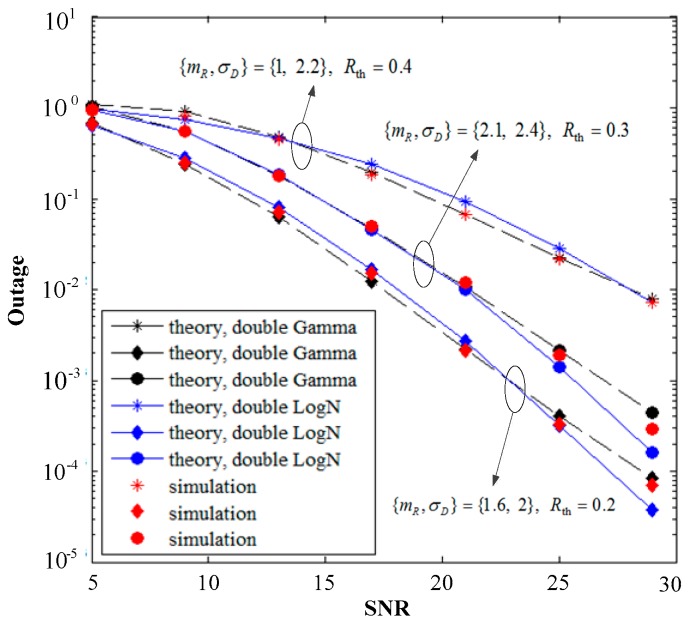
Comparison of analytical and simulated outage probabilities for various values of *R*_th_ thresholds.

Figure 7 shows the simulation and theoretical comparison of the system outage probability and the average SNR of the two approximated algorithms under different thresholds *R*_th_. The setting of the three sets of fading parameters is the same as that in Figure 6, and the Bernoulli-Gaussian noise parameter is the default setting. The three sets of curves in the figure show that the outage probability of the different parameters increases continuously as the value increases from 0.2 to 0.4, and the theoretical curves of the two algorithms are extremely close to the simulation results, thus verifying the correctness of our analysis. Furthermore, the curve of the L2G algorithm almost coincides with the simulation curve and the accuracy is higher than that of the G2L algorithm, however, the coincidence degree is slightly reduced at high SNR. Figure 7 shows that the algorithm can obtain sufficient computational accuracy in a certain range of SNR based on the consumption complexity.

**Figure 8:**
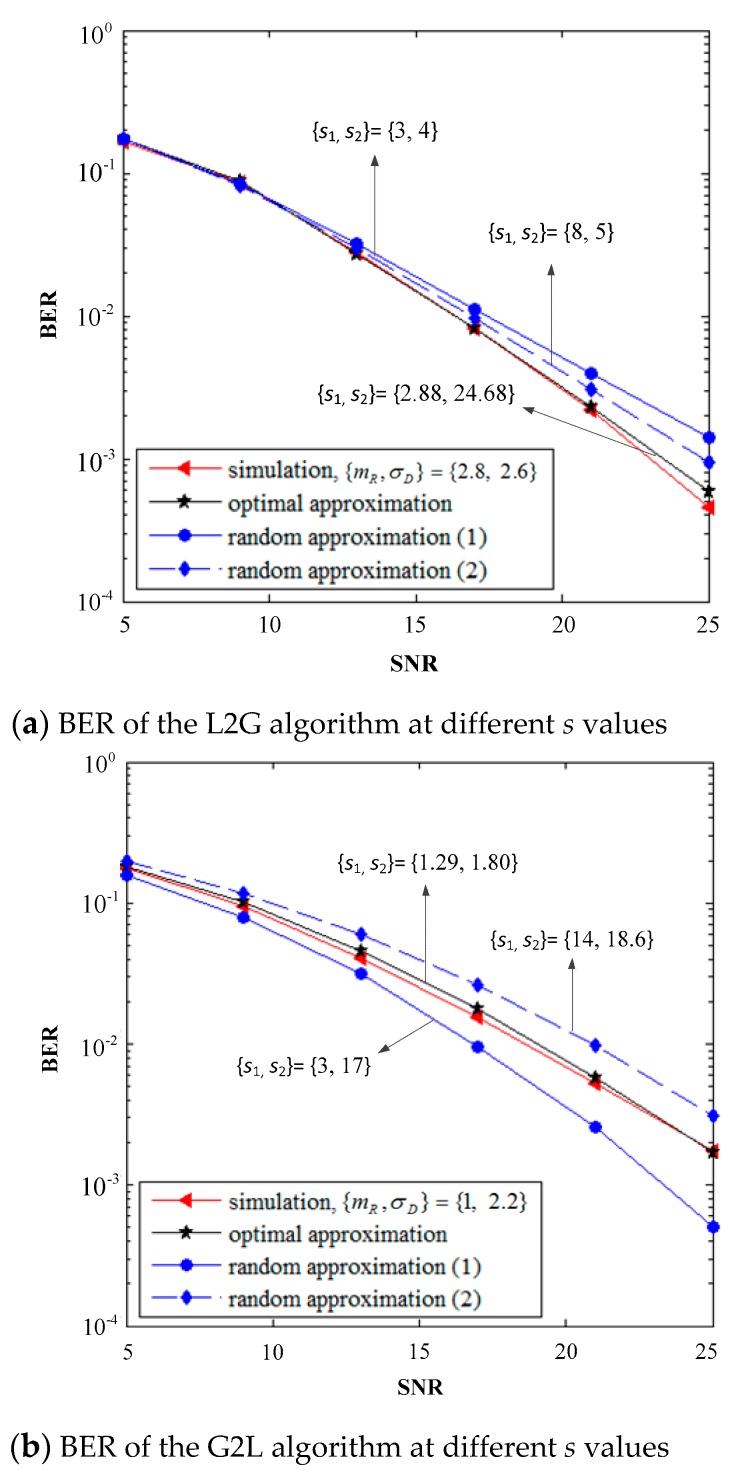
Influence of the selection of the *s* value in the two approximation algorithms on the system BER.

Figure 8 illustrates the performance of BER in the two approximation algorithms with the different approximation accuracies caused by the value of the *s* vectors. The fading parameters are $\{m_{R},\sigma_{D}\}=\{2.8,\;2.6\}{,}_{}\{1,\;2.2\}$, and the other parameters are set by default. Figure 8a,b shows that the coincidence between the theoretical algorithms and the simulation of the two approximation theoretical algorithms with the best *s* values is much higher than that with the random selection of *s* values. This outcome proves that the selection of the best *s* values is more conducive to obtaining the fading parameters of the approximate distribution accurately in the approximation process based on the PDF and MGF equations, wherein the system performance can be analyzed more accurately.

**Figure 9:**
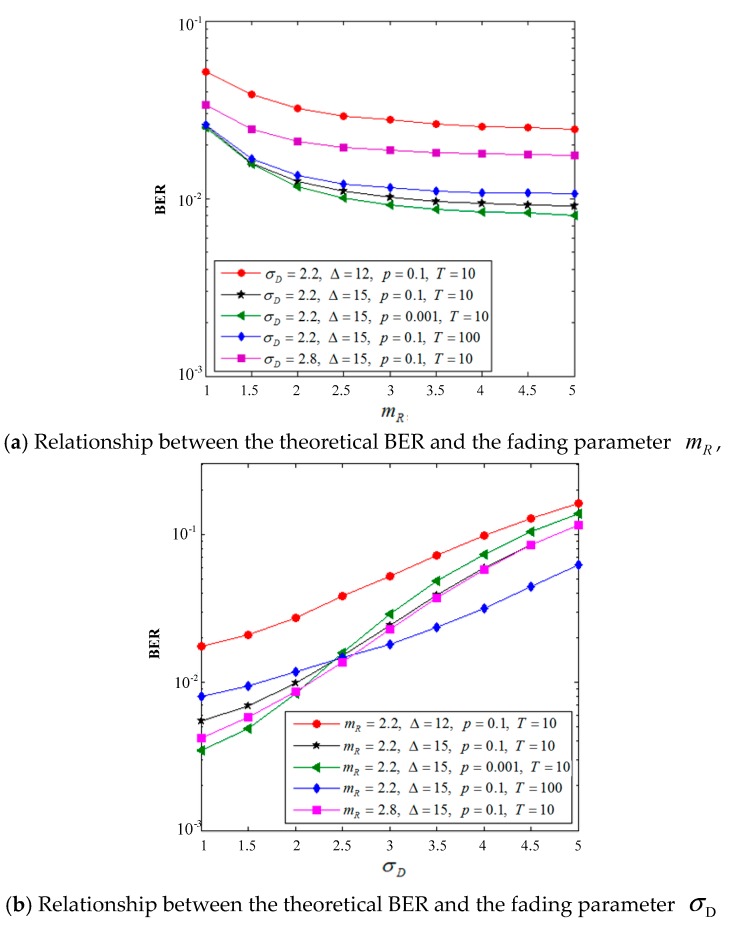
Comparison of the system analytical BER and the fading parameters $m_{R}^{}$ and $\sigma_{D}^{}$ under various modulation schemes.

The two graphs in Figure 9 compare the relationship of the system BER with the fading parameters $m_{R}$ and $\sigma_{D}$ under different channel parameter settings. We assume here that the fading parameters are 2.2 and 2.8 and that the average SNR Δ values are 12 and 15 dB, respectively. To highlight the contrast, *p* and *T* have two additional values of 0.001 and 100 respectively in addition to the initial default values. For the horizontal and vertical analyses of Figure 9, the following conclusions can be drawn: (1) Figure 9a shows that the system BER decreases as the $m_{R}$ increases (the wireless link fading is reduced), while the other parameters remain unchanged. When $m_{R}$ is larger than 2.5, the BER curve gradually flattens and the influence on the BER performance decreases. Similarly, Figure 9b shows that the BER performance of the system increases with the increase of $\sigma_{D}$ (serious power line link fading) under the condition that other parameters are constant, and the change trend of the BER curve is more significant than that of Figure 9a. Therefore, we conclude that the BER performance of the system is more sensitive to the selection of $\sigma_{D}$ than that of $m_{R}$. Therefore, for the dual-hop hybrid fading system, the LogN link occupies the dominant link, and the system performance can be optimized by significantly adjusting the environment variables of the link. (2) Figure 9a,b shows that the change of the channel noise parameters has a small impact on the BER. For example, when other parameters remain unchanged, the system performance improves slightly after *p* changes from 0.1 to 0.001, and the BER changes slightly after *T* changes from 10 to 100. (3) When the fading parameters *p* and *T* are the same, the BER performance of the system will significantly improve by increasing Δ, as reflected in both graphs. However, the improvement of the system will be different by increasing the same Δ under different fading parameters.

## 5. Conclusions

In this study, a novel type of dual-hop wireless power line hybrid communication system based on the AF relay protocol is analyzed. The proposed system uses the hybrid channel fading based on the Nakagami-m and LogN distributions, and the Bernoulli-Gaussian noise model is used for the mathematical modeling and theoretical deduction and analysis of the system. The difficulty in analyzing the system performance increases because the PDF at the end of the hybrid fading system is difficult to solve. Therefore, the MGF equation based on PDF is used to transform the performance analysis of the hybrid fading system into the performance analysis of the double Gamma fading system and further derive the precise closed-form expressions of the outage probability and BER of the system. At the same time, we execute the process of G2L, and the performance indicators of the double LogN distribution system are analyzed by using the approximation of the LogN variable sum, integral change, Mehta, and other algorithms. The numerical results show that the accuracy of the L2G algorithm is higher than that of the G2L algorithm. However, the L2G algorithm is also sensitive to parameter selection because of its high complexity. The G2L algorithm is beyond the limits of the MGF and can be used to analyze many performance indicators. The fading and noise parameters of each link can affect the system performance to varying degrees. Hence, the LogN distribution link is crucial in the entire system, and the combination of environmental variables of the link can be adjusted to optimize the system performance.

## Figures and Tables

**Table 1:** Optimal solution for different power line fading parameters *σ*_D._

*σ*_D_	*s*_1_	*s*_2_	*m*_D_	β_D_	Objective Function
2	3.2051	23.6809	4.2187	0.1997	2.0254
2.5	2.8908	24.6753	3.4932	0.2339	1.0062
2.8	2.8617	22.8037	3.1355	0.2561	0.6931
3	3.0924	17.5526	2.8639	0.2776	0.6951
3.3	3.2179	15.1707	2.5934	0.3015	0.7413

**Table 2:** Optimal solution for different wireless line fading parameters *m*_R._

m_R_	s_1_	s_2_	$\mu_{R}$	$\sigma_{R}$	Objective Function
2	3.7589	14.7629	−0.0852	0.4554	0.8255
2.5	4.1872	21.2461	−0.0472	0.4197	0.6104
2.9	2.4379	15.7817	−0.0416	0.4005	0.5272
3.2	2.3628	20.4712	−0.0340	0.3854	0.5281
3.5	2.9697	20.3867	−0.0178	0.3700	0.7707

## Appendix A

**Proof.** Note that $M_{Z}(s;\alpha_{R},\alpha_{D},\beta_{R},\beta_{D})=\int_{0}^{\infty }\Omega \left(s,t\right)dt-\int_{1}^{\infty }\Omega \left(s,t\right)dt$. The right integral $\int_{1}^{\infty }\Omega \left(s,t\right)dt$ is transformed first using t = 1 + x/s. The right integral is then presented as

(A1) $$\int_{0}^{\infty }\frac{\beta_{R}^{\alpha_{D}}\cdot \beta_{D}^{\alpha_{R}}\cdot {\left(1+\frac{x}{s}\right)}^{\alpha_{D}-1}{\left(-\frac{x}{s}\right)}^{\alpha_{R}-1}\cdot \frac{1}{s}}{B(\alpha_{R},\alpha_{D})\cdot {\left(\beta_{R}^{}+\left(\frac{\beta_{R}^{}-\beta_{D}^{}}{s}-\beta_{R}^{}\beta_{D}^{}\right)x-\frac{\beta_{R}^{}\beta_{D}^{}}{s}x^{2}\right)}^{\alpha_{R}+\alpha_{D}}}dx\overset{s\to \infty }{\approx }\int_{0}^{\infty }\frac{\beta_{R}^{\alpha_{D}}\cdot \beta_{D}^{\alpha_{R}}\cdot \frac{1}{s}\cdot {\left(-\frac{x}{s}\right)}^{\alpha_{R}-1}}{B(\alpha_{R},\alpha_{D})\cdot {\left(\beta_{R}^{}-\beta_{R}^{}\beta_{D}^{}x\right)}^{\alpha_{R}+\alpha_{D}}}dx,$$

with the aid of [30], Equation (A1) can be simplified as

(A2) $$\int_{1}^{\infty }\Omega \left(s,t\right)dt\overset{s\to \infty }{\approx }-s^{-\alpha_{R}}\beta_{R}^{-\alpha_{R}}.$$

Similarly, by using *t* = *x*/*s*, the left integral $\int_{0}^{\infty }\Omega \left(s,t\right)dt$ in the MGF can be transformed into

(A3) $$\int_{0}^{\infty }\Omega \left(s,t\right)dt\overset{s\to \infty }{\approx }s^{-\alpha_{D}}\beta_{D}{}^{-\alpha_{D}}.$$

As s→∞, |*M_γ_*($s;\alpha_{R},\alpha_{D},\beta_{R},\beta_{D}$)| = *b*|*s*|*^−d^* + *o*(|*s*|^−d^), then the diversity order for the relaying system is *d* [31]. Based on Equations (A2) and (A3), we have $M_{Z}\left(s;\alpha_{R},\alpha_{D},\beta_{R},\beta_{D}\right)\approx s^{-\alpha_{D}^{}}\beta_{D}{}^{-\alpha_{D}}+s^{-\alpha_{R}}\beta_{R}{}^{-\alpha_{R}}$ (as s→∞), and the diversity order is min(*α*_R_, *α*_D_) for the system model in this study. □

## References

[1] Dubey A., Mallik R.K. (2015). PLC system performance with AF relaying. IEEE Trans. Commun..

[2] Ferreira H.C., Lampe L., Newbury J., Swart T.G. (2011). Power Line Communications: Theory and Applications for Narrowband and Broadband Communications over Power Lines.

[3] Guzelgoz S., Celebi H.B., Arslan H. (2011). Statistical characterization of the paths in multipath PLC channels. IEEE Trans. Power Deliv..

[4] Cho W., Cao R., Yang L. (2008). Optimum resource allocation for amplify-and-forward relay networks with differential modulation. IEEE Trans. Signal Process..

[5] Papaleonidopoulos I.C., Capsalis C.N., Karagiannopoulos C.G., Theodorou N.J. (2003). Statistical analysis and simulation of indoor singlephase low voltage power-line communication channels on the basis of multipath propagation. IEEE Trans. Consum. Electron..

[6] Hong Y.W.P., Jen H.W., Kuo C.C.J. (2010). Cooperative Communications and Networking: Technologies and System Design.

[7] Zou H., Chowdhery A., Jagannathan S., Cioffi J.M., Masson J.L. Multi-user joint subchannel and power resource-allocation for power line relay networks. Proceedings of the 2009 IEEE International Conference on Communications.

[8] Tan B., Thompson J. Relay transmission protocols for in-door power line communications networks. Proceedings of the 2011 IEEE International Conference on Communications Workshops (ICC).

[9] Balakirsky V.B., Vinck A.J.H. Potential performance of PLC systems composed of several communication links. Proceedings of the International Symposium on Power Line Communications and Its Applications.

[10] Lampe L., Schober R., Yiu S. (2006). Distributed space-time coding for multihop transmission in power line communication networks. IEEE J. Sel. Areas Commun..

[11] Dubey A., Mallik R.K., Schober R. (2015). Performance analysis of a multihop power line communication system over log-normal fading in presence of impulsive noise. IET Commun..

[12] Lampe L., Vinck A.J.H. (2011). On cooperative coding for narrow band PLC networks. AEU Int. J. Electron. Commun..

[13] Rabie K.M., Adebisi B., Tonello A.M., Nauryzbayev G. (2018). For more energy-efficient dual-hop DF relaying power-line communication systems. IEEE Syst. J..

[14] Lai S.W., Messier G.G. The wireless/power-line diversity channel. Proceedings of the 2010 IEEE International Conference on Communications.

[15] Chen Z., Han D., Qiu L. (2017). Research on performance of indoor wireless and power line dual media cooperative communication system. Proc. CSEE.

[16] Mathur A., Bhatnagar M.R., Panigrahi B.K. Performance of a dual-hop wireless-powerline mixed cooperative system. Proceedings of the 2016 International Conference on Advanced Technologies for Communications (ATC).

[17] Lai S.W., Messier G.G. (2012). Using the wireless and PLC channels for diversity. IEEE Trans. Commun..

[18] Sarafi A.M., Tsiropoulos G.I., Cottis P.G. (2009). Hybrid wireless broadband over power lines: A promising broadband solution in rural areas. IEEE Commun. Mag..

[19] Zimmermann M., Dostert K. (2002). Analysis and modeling of impulsive noise in broad-band power line communications. IEEE Trans. Electromagn. Compat..

[20] Herath S.P., Tran N.H., Lengoc T. (2015). Optimal signaling scheme and capacity limit of PLC under Bernoulli-Gaussian impulsive noise. IEEE Trans. Power Deliv..

[21] Mehta N.B., Wu J., Molisch A.F., Zhang J. (2007). Approximating a sum of random variables with a lognormal. IEEE Trans. Wirel. Commun..

[22] Chen Z., Jing Y., Han D. (2018). Power optimization allocation based on approximation of moment generator function in dual media cooperative communication. Autom. Electr. Power Syst..

[23] Chu S.I. (2011). Performance of amplify-and-forward cooperative communications with the N^{th} best-relay selection scheme over Nakagami-m fading channels. IEEE Commun. Lett..

[24] Yang J., Fan P., Duong T.Q., Lei X. (2011). Exact performance of two-way AF relaying in Nakagami-m fading environment. IEEE Trans. Wirel. Commun..

[25] Ikki S.S., Ahmed M.H. (2009). Performance of cooperative diversity using Equal Gain Combining (EGC) over Nakagami-m fading channels. IEEE Trans. Wirel. Commun..

[26] Xia M., Aissa S. (2012). Moments based framework for performance analysis of one-way/two-way CSI-assisted AF relaying. IEEE J. Sel. Areas Commun..

[27] Li J., Qian X., Ge J., Zhang C. (2014). General and efficient relay selection for two-way opportunistic amplify-and-forward relaying. Electron. Lett..

[28] Guzelgoz S., Celebi H.B., Arsian H. Analysis of a multi-channel receiver: Wireless and PLC reception. Proceedings of the European Signal Processing Conference.

[29] Hasna M.O., Alouini M.S. (2004). Harmonic mean and end-to-end performance of transmission systems with relays. IEEE Trans. Commun..

[30] Gradshteyn I.S., Ryzhik I.M. (2007). Table of Integrals, Series, and Products.

[31] Wang Z., Giannakis G.B. (2003). A simple and general parameterization quantifying performance in fading channels. IEEE Trans. Commun..

